# Hempseed (*Cannabis sativa*) Peptides WVSPLAGRT and IGFLIIWV Exert Anti-inflammatory Activity in the LPS-Stimulated Human Hepatic Cell Line

**DOI:** 10.1021/acs.jafc.1c07520

**Published:** 2022-01-10

**Authors:** Ivan Cruz-Chamorro, Guillermo Santos-Sánchez, Carlotta Bollati, Martina Bartolomei, Jianqiang Li, Anna Arnoldi, Carmen Lammi

**Affiliations:** † Department of Pharmaceutical Sciences, University of Milan, 20133 Milan, Italy; ‡ Departamento de Bioquímica Médica y Biología Molecular e Inmunología, Universidad de Sevilla, 41009 Seville, Spain

**Keywords:** food peptides, hempseed, inflammation, oxidative stress, NF-κB

## Abstract

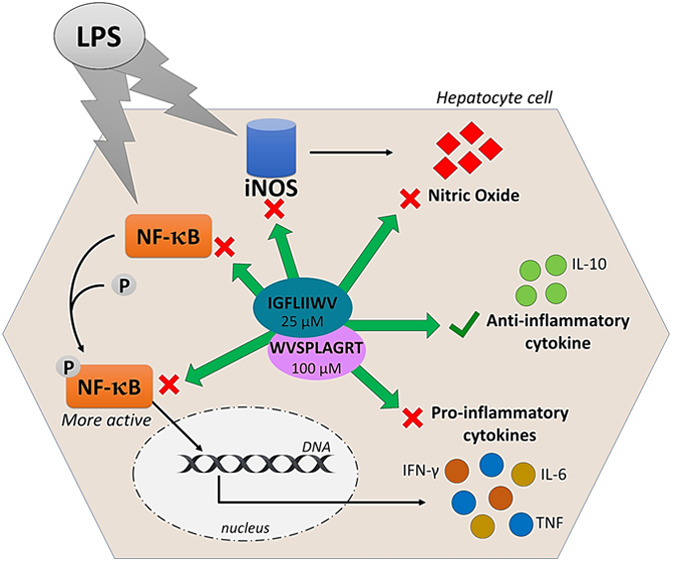

WVSPLAGRT (H2) and IGFLIIWV (H3) are two transepithelial transported intestinal peptides obtained from the hydrolysis of hempseed protein with pepsin, which exert antioxidant activity in HepG2 cells. Notably, both peptides reduce the H_2_O_2_-induced reactive oxygen species, lipid peroxidation, and nitric oxide (NO) production levels in HepG2 cells via the modulation of the nuclear factor erythroid 2-related factor 2 and the inducible nitric oxide synthase (iNOS) pathways, respectively. Due to the close link between inflammation and oxidative stress and with the objective of fostering the multifunctional behavior of bioactive peptides, in this study, the molecular characterization of the anti-inflammatory and immunomodulatory properties of H2 and H3 was carried out in HepG2 cells. In fact, both peptides were shown to modulate the production of pro (IFN-γ: −33.0 ± 6.7% H2, *p* = 0.011; −13.1 ± 2.0% H3, *p* = <0.0001; TNF: −17.6 ± 1.7% H2, *p* = 0.0004; −20.3 ± 1.7% H3, *p* = <0.0001; and IL-6: −15.1 ± 6.5% H3, *p* = 0.010)- and anti (IL-10: +9.6 ± 3.1% H2, *p* = 0.010; +26.0 ± 2.3% H3, *p* = < 0.0001)-inflammatory cytokines and NO (−9.0 ± 0.7% H2, *p* = <0.0001; −7.2 ± 1.8% H3, *p* = <0.0001) through regulation of the NF-κB and iNOS pathways, respectively, in HepG2 cells stimulated by lipopolysaccharides.

## Introduction

*Cannabis sativa* L. is a plant belonging to the *Cannabis* genus that has been used for medicinal purposes for hundreds of years.^1,2^ Its species present different levels of Δ9-tetrahydrocannabinol, the main psychoactive component that causes cognitive effects and euphoria.^3^ The non-drug variety (also called “hemp”’) is successfully used for industrial food industrial applications (i.e., nutritional supplements, fiber, and oil production) due to its quality nutritional composition.^4,5^ The hempseed is characterized by its high protein (20–25%) and oil content (more than 30%) as well as a complete profile of vitamins and minerals.^4^ Furthermore, its proteins (principally edestin and albumin) are easily digested and rich in essential amino acids, making hempseed an important source of bioactive peptides.^6,7^ Indeed, extensive studies have been carried out in order to investigate the multifunctional bioactive properties of hempseed peptides,^4^ demonstrating their antioxidant,^8−13^ hypotensive,^12,14,15^ antiproliferative,^16^ anti-inflammatory,^17,18^ and neuroprotective properties.^19^

Recently, our group has shown that hempseed hydrolysates (HP) produced to digest total protein with pepsin have a hypocholesterolemic effect through the direct ability to reduce the activity of the 3-hydroxy-3-methylglutaryl-coenzyme A reductase enzyme,^20,21^ which in turn leads to the activation of the low-density lipoprotein (LDL) receptor with the following improvement in the hepatic cells’ ability to absorb extracellular LDL.^20^ In addition, HP reduces the activity of the dipeptidyl peptidase-IV (DPP-IV) in vitro and in human intestinal Caco-2 cells, suggesting a potential anti-diabetic effect.^21^

Recent experiments using intestinal trans-epithelial transport revealed that among the peptides contained within HP able to pass through the mature Caco-2 cell barrier, H2 (WVSPLAGRT) and H3 (IGFLIIWV) exert antioxidant activity in HepG2 cells. Specifically, we observed that H2 and H3 reduce the level of reactive oxygen species (ROS), lipid peroxidation, and nitric oxide (NO) production. Furthermore, H2 and H3 modulate the nuclear factor erythroid 2-related factor 2 (Nrf-2) and inducible nitric oxide synthase (iNOS) pathways in H_2_O_2_-stimulated HepG2 cells.^22^

In light of these observations and considering that there is a close link between inflammation and oxidative stress, the main objective of the present study was the evaluation of the anti-inflammatory effect of peptides H2 and H3 in HepG2 cells. Therefore, since the nuclear factor-κB (NF-κB) pathway is the main component implicated in the pro-inflammatory response,^23^ the effects of H2 and H3 on the NF-κB and its more active phosphorylated form (p(Ser276)NF-κB) protein levels in lipopolysaccharide (LPS)-stimulated HepG2 cells were characterized at a deeper level. Hence, the effect of both peptides on the modulation of the cellular pro (IFN-γ, TNF, and IL-6)- and anti (IL-10)-inflammatory cytokine production was evaluated. Finally, in parallel, the effects of H2 and H3 on the NO pathway, which plays a central role in inflammatory disorders,^24^ were investigated.

## Materials and Methods

### Chemicals and Reagents

All reagents and solvents were purchased from commercial sources and used without further purification. For further details, see theSupporting Information. The peptides were purchased from GenScript Biotech Corporation (Piscataway, NJ, USA). The purity of lyophilized peptides (>95%) was tested using a binary high-performance liquid chromatograph and an Agilent 6520 LCMS mass spectrometer (Figure S1).

### Cell Culture and Western Blot

A total of 1.5 × 10^5^ HepG2 cells/well were seeded in 24-well plates and incubated at 37 °C under a 5% CO_2_ atmosphere. The following day, the cells were stimulated with 1 μg/mL LPS or vehicle (H_2_O) and treated with 100 μM H2 or 25 μM H3 peptides in a complete growth medium (10% fetal bovine serum, 100 U/mL penicillin, and 100 μg/mL streptomycin) for another 48 h. Finally, the supernatant was collected and stored at −20 °C for subsequent cytokine and NO quantification.

The cells were scraped in 40 μL of ice-cold lysis buffer (RIPA buffer + protease inhibitor cocktail (Roche, Base, Swiss) + 1:100 PMSF + 1:100 Na-orthovanadate + 1:1000 β-mercaptoethanol) and transferred to ice-cold microcentrifuge tubes. After centrifugation at 13,300 g for 15 min, the supernatants were recovered for Western Blot analysis. The total protein concentration was determined by Bradford’s method. 50 μg of proteins was separated on a precast 7.5% sodium dodecyl sulfate-polyacrylamide gel in the presence of a reducing agent (β-mercaptoethanol), transferred to a nitrocellulose membrane (Mini nitrocellulose Transfer Packs, Biorad, Hercules, CA, USA), and stained with Ponceau red solution. Later, milk/BSA blocked membranes were incubated with primary antibodies against iNOS, NF-κB, phosphor(Ser276)-NF-κB (p(Ser276)NF-κB), and β-actin (more details in Supporting Information Table S1). Membranes were incubated overnight at 4 °C and consequently with the horseradish peroxidase-conjugated secondary antibody. Finally, target proteins were detected with enhanced chemiluminescence (Euroclone, Milan, Italy), and densitometric analysis was performed using Image Lab Software (Biorad).

### Cytokine Quantification

Cytokine quantification was performed using a human Quantikine ELISA kit (R&D Systems, Minneapolis, MN, USA) according to the manufacturer’s instructions. Briefly, the supernatant was incubated in 96-well microplates coated with a monoclonal antibody for 2 h. After washing the wells, the human polyclonal antibody conjugated with horseradish peroxidase was added for another 2 h. The wells were washed, and then a substrate solution was added to obtain a color. The reaction was stopped by a stop solution (2 N sulfuric acid), and then the microplate was read to wavelength 450 nm and 540 nm with a Synergy H1 microplate reader (Biotek Instruments, Winooski, VT, USA).

### Nitric Oxide Quantification

NO determination was quantified in the supernatants by the Griess test (Sigma-Aldrich, Milan, Italy) according to the manufacturer’s instructions. Briefly, 50 μL of the Griess reagent was incubated with 50 μL of the culture supernatants for 15 min at room temperature in the dark. Absorbance at 540 nm was then measured using a Synergy H1 microplate reader (Biotek).

### Statistical Analysis

The data were presented as the mean ± the standard deviation (SD) of at least three independent experiments assayed for triplicate. All the data sets were checked for normal distribution by the D’Agostino and Pearson test. Since they are all normally distributed with *p*-values <0.05, we proceeded with statistical analyses by one-way ANOVA, followed by Tukey’s post-hoc tests and using GraphPad Prism 8 (San Diego, CA, USA).

## Results

### H2 and H3 Modulate the LPS-Activated NF-κB Pathway in HepG2 Cells

**Figure 1:**
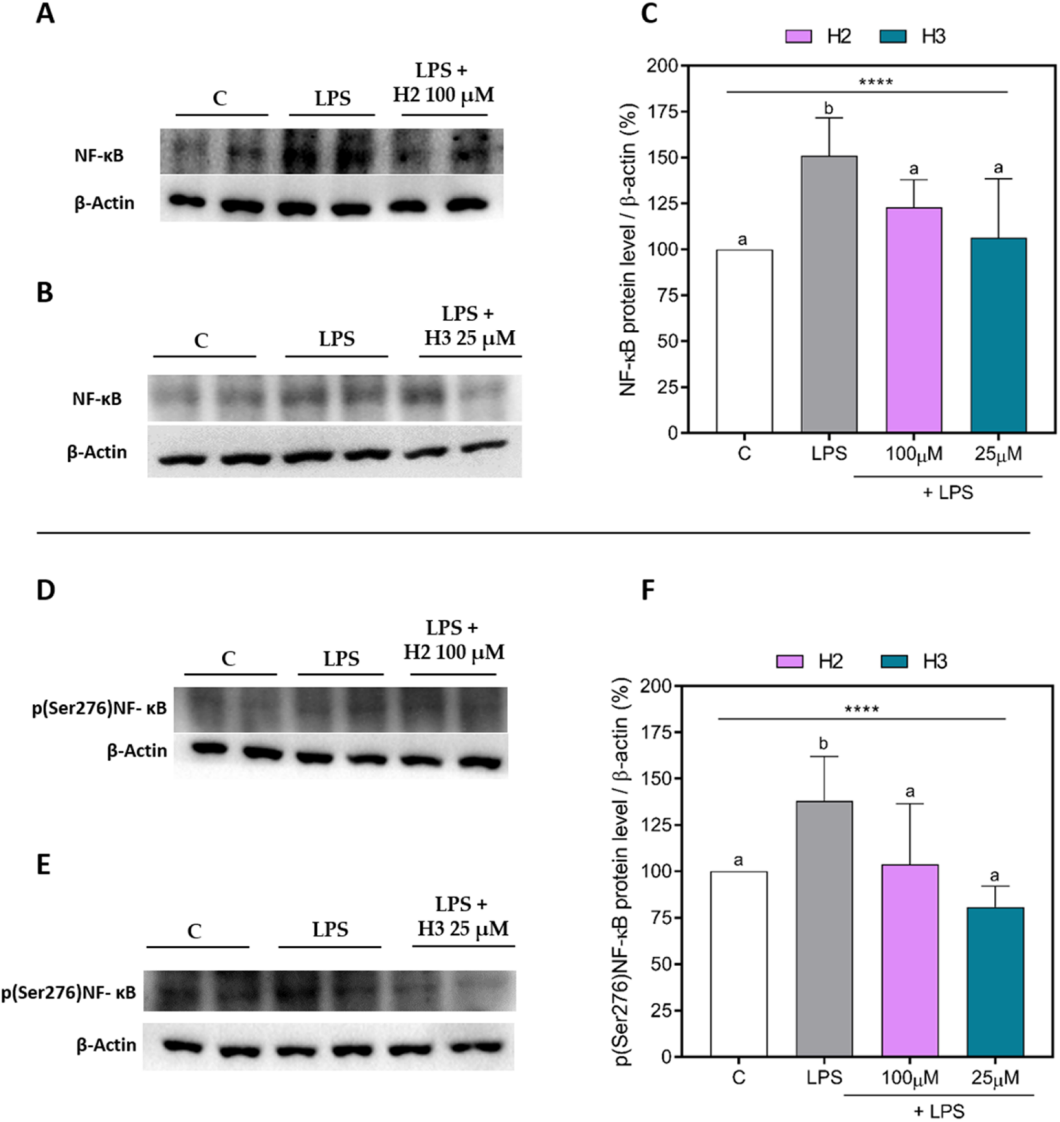
NF-κB and p(Ser276)NF-κB protein levels in LPS-stimulated HepG2 cells. Upper panel: representative Western Blots of NF-κB in H2 (A) and H3 (B) assays. Densitometric analyses of NF-κB (C). Bottom panel: representative Western Blots of p(Ser276)NF-κB in H2 (D) and H3 (E) assays. Densitometric analyses of p(Ser276)NF-κB (F). The data points represent the averages ± SD of three independent experiments in triplicate. All data sets were analyzed by one-way ANOVA, followed by Tukey’s post-hoc test. Different letters indicate statistically significant differences. ****, *p* ≤ 0.0001. C, unstimulated control group; LPS, lipopolysaccharide-stimulated cells; NF-κB, nuclear factor-κB; p(Ser276)NF-κB, phosphor(Ser276)-nuclear factor-κB.

**Figure 2:**
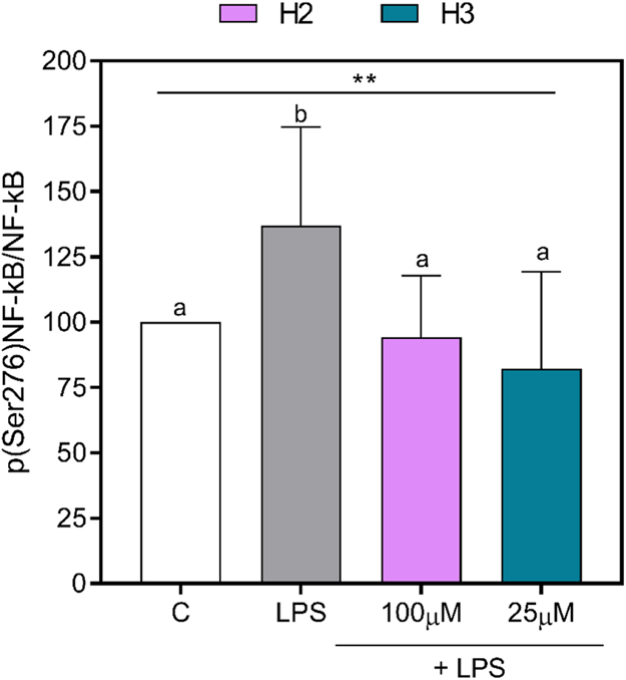
p(Ser276)NF-κB/NF-κB ratio. The histogram represents the averages ± SD of the p(Ser276)NF-κB/NF-κB ratios of three independent experiments in triplicate. All data sets were analyzed by one-way ANOVA, followed by Tukey’s post-hoc test. Different letters indicate statistically significant differences. **, *p* ≤ 0.01. C, unstimulated control group; LPS, lipopolysaccharide-stimulated cells; NF-κB, nuclear factor-κB; p(Ser276)NF-κB, phosphor(Ser276)-nuclear factor-κB.

To investigate the effects of H2 and H3 on the NF-κB pathway, NF-κB and p(Ser276)NF-κB were quantified in LPS-stimulated HepG2 cells. As shown in Figure 1, the LPS stimulation confirmed the NF-κB pathway activation, increasing the protein levels of NF-κB (Figure 1A–C) and p(Ser276)NF-κB (Figure 1D–F) in HepG2 cells up to 150.9 ± 20.7% (*p* < 0.0001) and 138.0 ± 24.1% (*p* = 0.0008), respectively. Treatment with H2 and H3 mitigated these effects. In detail, H2 (violet bars) significantly reduced the NF-κB protein levels by 28.1 ± 5.5% (*p* = 0.034) at 100 μM, with respect to LPS-stimulated cells (Figure 1A,C). Peptide H3 (aquamarine bar) showed a similar effect, reducing the NF-κB levels by up to 44.3 ± 11.2% (*p* = 0.002) at 25 μM (Figure 1B,C). In addition, both peptides were able to decrease the more active phosphorylated form of NF-κB (Figure 1D–F). H2 was able to reduce the p(Ser276)NF-κB levels by 34.1 ± 8.5% (*p* = 0.013) at 100 μM (Figure 1D,F), while H3 decreased the levels by 57.2 ± 13.0% (*p* = 0.0002) at 25 μM (Figure 1E,F). As shown in Figure 2, LPS treatment increased the p(Ser276)NF-κB/NF-κB ratio up to 137 ± 37.7% (*p* = 0.039), underlining more activation of NF-κB, while both peptides were able to decrease this ratio, confirming that these peptides promoted less activation of this pathway. Specifically, H2 decreased the p(Ser276)NF-κB/NF-κB ratio by 42.9 ± 14.0% (*p* = 0.046) at 100 μM, while H3 reduced this ratio by 54.7 ± 0.7% (*p* = 0.014) at 25 μM (Figure 2).

### H2 and H3 Decrease the LPS-Induced Cytokine Production in Hepatic HepG2 Cells

**Figure 3:**
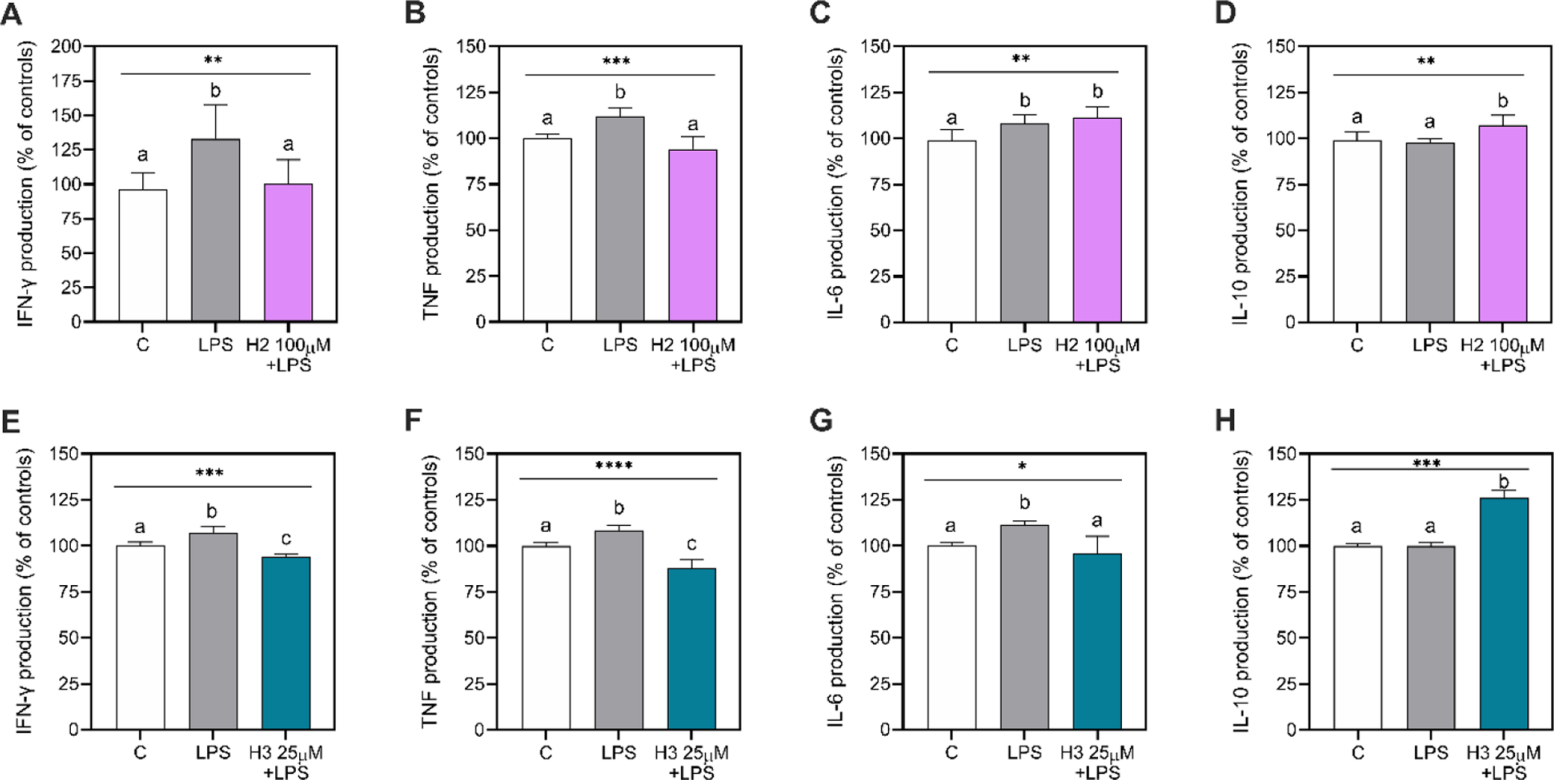
Cytokine production in HepG2 cells. Pro-inflammatory (A–C, E–G) and anti-inflammatory (D,H) cytokines. Data presented as mean ± SD of three independent experiments performed in triplicate. All data sets were analyzed by one-way ANOVA, followed by Tukey’s post-hoc test. *, *p* ≤ 0.05; **, *p* ≤ 0.01; ***, *p* ≤ 0.001; ****, *p* ≤ 0.0001. Different letters indicate statistically significant differences. C, unstimulated control group; IFN-γ, interferon-γ; IL, interleukin; LPS, lipopolysaccharide-stimulated cells; TNF, tumor necrosis factor.

To verify the possible immune effect of the two peptides, the influence of treatment with H2 (100 μM) or H3 (25 μM) on the production of pro-inflammatory (IFN-γ, TNF, IL-6) and anti-inflammatory (IL-10) cytokines was determined in LPS-stimulated HepG2 cell culture supernatants. As shown in Figure 3, the LPS stimulation increased the production of the pro-inflammatory cytokines (Figure 3A–C,E–G), without affecting the IL-10 production (Figure 3D,H), compared with LPS-unstimulated and untreated cells (control, C). Indeed, H2 successfully restored the normal concentrations of IFN-γ and TNF. In detail, H2 reduced by 33.0 ± 6.7% (*p* = 0.011) and 17.6 ± 1.7% (*p* = 0.0004) the LPS-induced IFN-γ and TNF production at 100 μM (Figure 3A,B), respectively. Despite this reduction, H2 was not able to alter the LPS-induced IL-6 production at the same concentration of 100 μM (*p* = 0.581) (Figure 3C). However, H2 increased the IL-10 production by 9.6 ± 3.1% at 100 μM (*p* = 0.010) compared to the LPS-stimulated cells (Figure 3D).

A similar scenario was also observed for the H3 peptide. In this case, anti-inflammatory effects were already observed at 25 μM (Figure 3E–H). In particular, H3 reduced IFN-γ and TNF production by 13.1 ± 2.0% (*p* = <0.0001) and 20.3 ± 1.7% (*p* = <0.0001), respectively, compared to LPS-stimulated cells (Figure 3E,F). In addition, unlike H2, H3 decreased IL-6 production by 15.1 ± 6.5% (*p* = 0.010) (Figure 3G), restoring the normal values as in LPS-unstimulated and untreated HepG2 cells (C). Surprisingly, H3 also increased the IL-10 production by 26.0 ± 2.3% (*p* = <0.0001) in comparison to the LPS-stimulated cells (Figure 3H).

Absolute values (mean ± SD) of the cytokine production are reported in Supporting Information Table S2.

### H2 and H3 Promote an Anti-inflammatory Microenvironment

In order to verify whether the peptides H2 and H3 were able to promote a more anti-inflammatory microenvironment, the anti- and pro-inflammatory cytokines’ ratio was calculated. As shown in Table 1, H2 was able to increase the anti-inflammatory microenvironment (IL-10/IFN-γ: 100 μM, *p* = 0.002; or IL-10/TNF: 100 μM, *p* = 0.0006), skewing this ratio to a higher IL-10 content, in comparison with the LPS-stimulated cells. Also, in this case, when the ratio of IL-10 with IL-6 was calculated, a significant alteration in their proportion was observed (H2 100 μM, *p* = 0.336).

**Table 1:** Anti-/Pro-inflammatory Cytokines’ Ratio^a^

	C	LPS	H2 [100μM]
IL10/IFN-γ	1.05 ± 0.14^a^	0.75 ± 0.15^b^	1.10 ± 0.19^a^
IL10/TNF	0.99 ± 0.07^a^	0.88 ± 0.05^a^	1.14 ± 0.11^b^
IL10/IL-6	1.01 ± 0.10^a^	0.90 ± 0.03^a^	0.96 ± 0.07^a^

	C	LPS	H3 [25μM]
IL10/IFN-γ	1.00 ± 0.03^a^	0.93 ± 0.05^a^	1.34 ± 0.06^b^
IL10/TNF	1.00 ± 0.02^a^	0.92 ± 0.02^a^	1.44 ± 0.12^b^
IL10/IL-6	1.00 ± 0.02^a^	0.90 ± 0.01^a^	1.32 ± 0.13^b^

a Ratios between anti-inflammatory (IL-10) and pro-inflammatory (IFN-γ, TNF, and IL-6) cytokines quantified in HepG2 cells stimulated or not with LPS and treated with H2 (100 μM) or H3 (25 μM). Data are presented as mean ± SD and were analyzed by one-way ANOVA, followed by Tukey’s post-hoc test. Different letters indicate statistically significant differences (*p* ≤ 0.05). C, unstimulated control group; IFN-γ, interferon-γ; IL, interleukin; LPS, lipopolysaccharide-stimulated cells; TNF, tumor necrosis factor.

In line with cytokine quantification, H3 showed an improvement in the proportion of IL-10 with respect to IFN-γ (*p* ≤ 0.0001), TNF (*p* ≤ 0.0001), and IL-6 (*p* ≤ 0.0001) at 25 μM, with respect to the LPS-stimulated cells’ group.

Since the differences in the IL-10/IL6 ratio were not detected with H2 treatment, we decided to verify if there existed a correlation between the IL-10 and IL-6 production under the different experimental conditions; thus, Pearson correlation was performed. As shown in Table 2, the negative correlation between these two cytokines in the LPS-stimulated condition was lost. On the contrary, H2 treatment restored a negative correlation between the anti-inflammatory IL-10 and pro-inflammatory IL-6 cytokine, as in the unstimulated and untreated control group (C).

**Table 2:** Pearson Correlation between IL-10 and IL-6 Production^a^

Pearson correlation	C	*p*-value	LPS	*p*-value	H2 [100μM]	*p*-value
IL-10 vs IL-6	–0.9239	**0.025**	–0.4795	0.414	–0.9628	**0.009**

a Data represent the Pearson r value obtained by the correlation between IL-10 and IL-6 production under the different experimental conditions.

### H2 and H3 Modulate the LPS-Activated iNOS Pathway in HepG2 Cells

**Figure 4:**
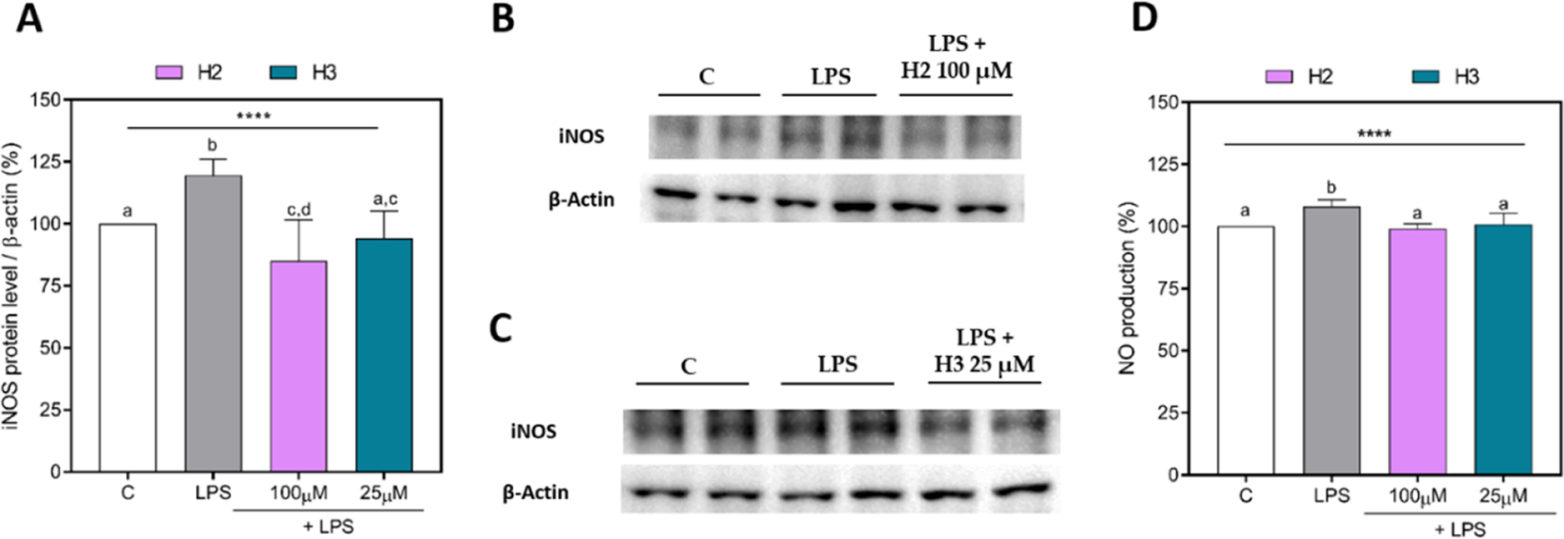
iNOS and NO production in HepG2 cells treated with H2 or H3. Densitometric analyses of iNOS protein levels (A); representative Western Blots of iNOS in H2 (B) and H3 (C) assays; NO production (D). The data points represent the averages ± SD of three independent experiments in triplicate. All data sets were analyzed by one-way ANOVA, followed by Tukey’s post-hoc test. Different letters indicate statistically significant differences. ****, *p* < 0.0001. C, unstimulated control group; iNOS, inducible nitric oxide synthase; LPS, lipopolysaccharide-stimulated cells; NO, nitric oxide.

As shown in Figure 4, LPS stimulation induced an inflammatory state in HepG2 cells, increasing the iNOS and NO levels’ production up to 119.6 ± 6.4% (*p* ≤ 0.0001) (Figure 4A–C) and 108.1 ± 2.7% (*p* ≤ 0.0001) (Figure 4D), respectively. The treatment with H2 or H3 showed a significant reduction in iNOS and NO production, whose values were close to the baseline values. Specifically, H2 reduced the iNOS protein by 34.4 ± 9.9% (*p* ≤ 0.0001) (Figure 4A,B) and NO production by 9.0 ± 0.7% (*p* ≤ 0.0001) at 100 μM (Figure 4D). Furthermore, H3 was able to reduce the iNOS protein by 25.3 ± 4.4% (*p* ≤ 0.0001) (Figure 4A,C) and NO production by 7.2 ± 1.8% (*p* ≤ 0.0001) 25 μM (Figure 4D).

## Discussion

Recently, we demonstrated that peptides H2 and H3 exert antioxidant activity in HepG2 cells, modulating both the Nrf-2 and iNOS pathways, which led to the reduction of cellular H_2_O_2_-induced ROS, NO, and lipid peroxidation levels.^22^ Since an increase in oxidative stress is always accompanied by an inflammatory process, it was interesting to study the immunomodulatory capacity of these two hempseed peptides in the same cellular system. Notably, HepG2 cells have been widely used as a model for characterizing the anti-inflammatory activity of many food-active compounds.^25−28^ To achieve this objective, we decided to perform tests under the same conditions and study the same concentration of each peptide H2 (100 μM) and H3 (25 μM), which was previously demonstrated to be safe from a cytotoxic point of view and also effective for antioxidant activity.^22^ In particular, HepG2 cells were stimulated with LPS, a generic and commonly used pro-inflammatory stimulus. Therefore, the LPS stimulation activates the NF-κB and iNOS pathways.^29^ NF-κB is the main transcription factor involved in all pro-inflammatory processes of the mammalian organism, and it mediates the pro-inflammatory cytokine transcription, such as IFN-γ, TNF, and IL-6.^23^ On the other hand, iNOS is involved in the immune response, producing NO, a free radical involved in the immune defense mechanism.^30^

In this work, we showed that hempseed hydrolysates can turn off pro-inflammatory signaling by modulating the NF-κB and iNOS pathways’ modulation. In fact, both H2 and H3 were able to decrease the NF-κB protein as well as its more active form phospho(Ser276)NF-κB. The p65 subunit of NF-κB contains the transactivation domain, which is involved in the driving of transcription.^31^ There are several mechanisms involved in the modulation of the NF-κB activity; therefore, the crosstalk with other signaling pathways allows one to act on the transactivating ability of NF-κB. For example, the NF-κB activity is favored by p38 mitogen-activated protein kinase, which phosphorylates the p65 subunit in the residue 276 serine.^32,33^ This phosphorylation allows interaction with other transcriptional co-activators, thus increasing the NF-κB activity. Thus, the decrease of phospho-Ser276-p65 observed with H2 and H3 treatment demonstrated their NF-κB activity inhibition capacity. In addition, both hydrolysates favored an anti-inflammatory microenvironment, skewing the ratio to the less active NF-κB form. To confirm this NF-κB inhibitory ability, the cytokine profile was studied. The results obtained showed that the NF-κB pathway was inhibited since a decrease in pro-inflammatory cytokines was observed. Moreover, a major proportion of anti-inflammatory IL-10 cytokine was observed with respect to the pro-inflammatory cytokines. IL-10 exerts many anti-inflammatory functions, and it is the principal cytokine involved in finishing the inflammation processes, such as inhibiting the NF-κB pathway, among others.^34^ Therefore, the increase in IL-10 production mediated by H2 and H3 is strongly related to the NF-κB pathway inhibition.

Although H2 was not able to alter the LPS-induced IL-6 production and the IL-10/IL-6 ratio, a negative correlation was observed. In fact, LPS stimulation altered the correlation between IL-10 and IL-6, while H2 treatment re-establishes this negative correlation, demonstrating that a major IL-10 concentration corresponds to less IL-6 production. These effects can be explained by the negative modulation of the NF-κB activity and then a less IL-6 production, although we did not observe significant differences by performing an ELISA assay.

Recently, hempseed hydrolysates obtained with Alcalase alone or in combination with Flavourzyme were shown to reduce the gene expression of TNF and IL-6, as well as increase IL-10 mRNA, in the LPS-stimulated BV2 microglia cell line.^19^ In addition, these same protein hydrolysates have been shown to reduce the production of inflammatory cytokines TNF, IL-6, and IL-1β as well as increase the anti-inflammatory cytokine IL-10 in primary human monocytes.^18^ However, no specific peptides were singled out as being responsible for this biological effect. Recently, it was demonstrated that two egg tripeptides (IRW and IQW) from ovotransferrin are effective in the down-regulation of cytokine-induced inflammatory protein expression in vascular endothelium, at least partially through the modulation of the NF-κB pathway.^35,36^ These two peptides are shorter than both H2 and H3; however, comparing their sequences with H2 and H3, it is feasible to consider that the Tryp and Ile presence may be positively correlated not only with the antioxidant but also with the anti-inflammatory effects, reinforcing the strong cross-linking between these two activities.^22,35^ Interestingly, the IRW and IQW beneficial effects require the presence of an intact tripeptide as the corresponding dipeptides and constituent amino acids alone failed to replicate the anti-inflammatory functions, indicating a structure–function relationship between the tripeptide structure and blockade of inflammation. A very interesting feature of both H2 and H3 is that despite IRW and IQW, they are transported by intestinal cells and they are stable toward intestinal protease activity when they are within the hempseed hydrolysate.^22^

Another interesting feature of our work is related to the ability evaluation of both H2 and H3 to modulate the iNOS pathway, which is known to be involved in immune response, producing NO, a free radical implicated in the immune defense mechanism.^30^

NO acts as a cytotoxic agent in pathological processes, specifically in inflammatory disorders.^37^ In this sense, numerous scientific articles have shown that NO production is elevated in chronic inflammatory diseases, such as diabetes,^38^ atherosclerosis,^39^ or multiple sclerosis.^40^ The iNOS protein is mainly responsible for the production of cellular NO;^24^ in fact, its inhibition may be a therapeutic target in inflammatory diseases.^41^ In our study, we observed that H2 and H3 peptides reduced NO and iNOS production in LPS-stimulated HepG2 cells. In addition, the reduction of NF-κB by peptides is also confirmed by the observed results in the NO pathways. NF-κB plays an important role in the regulation of iNOS production, inducing its expression,^42^ and, at the same time, it is well known that NO, in turn, can induce NF-κB activation.^38^ Our findings suggest, together with those that we have previously observed, a potential interplay of both antioxidant and anti-inflammatory activities exerted by H2 and H3 peptides. Moreover, the present study confirms that H3 is fourfold more active than H2 not only as an antioxidant but also as an anti-inflammatory peptide. Taking together all the results and on the basis on our knowledge, this study is the first to observe the role of two specific peptides in the regulation of the NO pathway in hepatic cells. In addition, although many food protein hydrolysates have demonstrated anti-inflammatory effects,^43^ our study is the pioneer in the identification of anti-inflammatory peptides that can be absorbed by the human intestinal barrier from the hempseed source.^22^

In conclusion, all these findings demonstrate that H2 and H3 peptides possess great anti-inflammatory capacity in the HepG2 cells. Both antioxidant^22^ and anti-inflammatory effects in HepG2 cells point out the useful employment of H2 and H3 how possible strategies to prevent liver diseases, such as non-alcoholic steatohepatitis, characterized by inflammation and oxidative stress in the early stages of the disease,^44^ even though dedicated in vivo study is necessary to confirm this important feature.

## Supporting Information Available

Chromatogram and mass spectrum of H2 and H3; antibodies used in the Western Blot assays; and absolute values of cytokine production (PDF)

## Abbreviations

BSA: bovine serum albumin
Caco-2: *Homo Sapiens* colorectal adenocarcinoma cells
DPP-IV: dipeptidyl peptidase-IV
HepG2: human hepatoma cells
H2: WVSPLAGRT hempseed peptide
H3: IGFLIIWV hempseed peptide
HP: peptic hempseed hydrolysate
IFN-γ: interferon-γ
IL: interleukin
iNOS: inducible nitric oxide synthase
LPS: lipopolysaccharide
NF-κB: nuclear factor-κB
NO: nitric oxide
PBS: phosphate buffered saline
ROS: reactive oxygen species
TNF: tumor necrosis factor
