# Supplementary material for: Hempseed (Cannabis sativa) Peptides WVSPLAGRT and IGFLIIWV Exert Anti-inflammatory Activity in the LPS-Stimulated Human Hepatic Cell Line
Source: J Agric Food Chem. 2022 Jan 10;70(2):577–83. doi: 10.1021/acs.jafc.1c07520 (PMC8778635; doi:10.1021/acs.jafc.1c07520)
Supplement: Supplementary file 1 — jf1c07520_si_001.pdf [file jf1c07520_si_001.pdf]

Supplemental material

The hempseed (C. sativa) peptides WVSPLAGRT and IGFLIIWV exert anti-inflammatory activity in LPS stimulated human hepatic cell line

Ivan Cruz-Chamorro [a,b,†], Guillermo Santos-Sánchez [a,b,†], Carlotta Bollati [a], Martina Bartolomei [a], Jianqiang Li [a], Anna Arnoldi [a], Carmen Lammi [a,*]

[a]Department of Pharmaceutical Sciences, University of Milan, 20133 Milan, Italy. carlotta.bollati@unimi.it (C.B.); martina.bartolomei@unimi.it (M.B.); anna.arnoldi@unimi.it (A.A.); jianqiang.li@unimi.it (J.L.); carmen.lammi@unimi.it (C.L.)

[b]Departamento de Bioquímica Médica y Biología Molecular e Inmunología, Universidad de Sevilla, 41009 Seville, Spain. icruz-ibis@us.es (I.C.-C.); gsantos-ibis@us.es (G.S.-S.)

† These authors equally contributed to the work

* Corresponding Author: Carmen Lammi, Department of Pharmaceutical Sciences, University of Milan, via Mangiagalli 25, 20133 Milan, Italy. E-mail: carmen.lammi@unimi.it, tel.: +39 02-50319372.

Reagents

Dulbecco's modified Eagle's medium (DMEM), L-glutamine, fetal bovine serum (FBS), phosphate buffered saline (PBS), penicillin/streptomycin, chemiluminescent reagent, and 24 or 96-well plates were purchased from Euroclone (Milan, Italy). Bovine serum albumin (BSA), RIPA buffer was bought from Sigma-Aldrich (St. Louis, MO, USA). Phenylmethanesulfonyl fluoride (PMSF), Na-orthovanadate inhibitors were purchased from Santa Cruz Biotechnology Inc. (Santa Cruz, CA, USA). The inhibitor cocktail Complete Midi from Roche (Basel, Swiss). Mini protean TGX pre-cast gel 7.5%, and Mini nitrocellulose Transfer Packs were purchased from BioRad (Hercules, CA, USA).

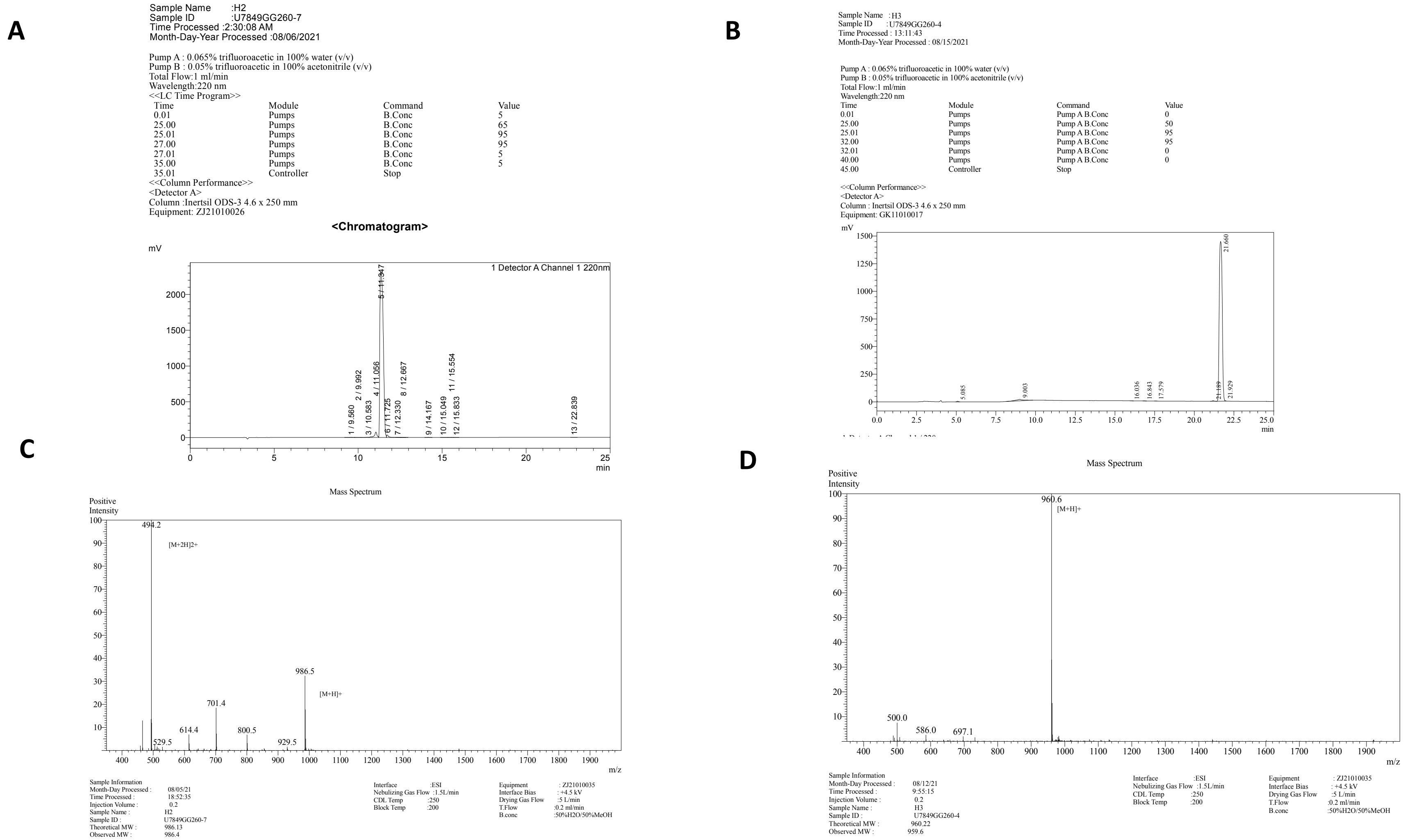

Figure S1: Chromatogram and Mass Spectrum of H2 (A and C) and H3 (B and D)

Table S1. Antibodies used in the Western Blot assays.

Name product	Dilution	Supplier
Anti-iNOS	1:1000	Cell Signaling Technology (D6B6S)
Anti-NF-kappaB, p65 subunit, active subunit, clone 12H11	1:1000	Sigma-Aldrich (MAB3026)
Anti-phospho-NF-kappaB p65 (pSer276)	1:1000	Sigma-Aldrich (SAB4504488)
Anti-β-actin	1:16000	Sigma-Aldrich (A5441)

Name of antibody, dilution used, and supplier. iNOS, inducible nitric oxide synthase; NF-kappaB, nuclear factor-κB.

Table S2. Absolute values of cytokine production.

Cytokine	C	LPS	H2 [100 μM]	C	LPS	H3 [25 μM]
IFN-γ (pg/mL)	25.50 ± 5.04	34.12 ± 5.94	26.00 ± 1.41	28.28 ± 1.28	30.35 ± 0.35	26.50 ±0.58
TNF (pg/mL)	33.71 ± 1.21	38.00 ± 2.94	32.00 ± 1.41	33.89 ± 0.75	36.68 ± 0.43	29.83 ± 1.17
IL-6 (ng/mL)	1329 ± 8.60	1448 ± 1.23	1470 ± 5.02	603.8 ± 1.71	672.5 ± 2.38	584.8 ± 19.16
IL-10 (pg/mL)	21.68 ± 0.22	20.98 ± 1.32	23.25 ± 0.13	48.90 ± 0.36	49.74 ± 1.43	61.88 ± 0.17

Mean ± standard deviation (SD) of the cytokines production. C, unstimulated control group; LPS, lipopolysaccharide-stimulated cells; H, hempseed peptide; IFN-γ, interferon-γ; TNF, tumor necrosis factor; IL, interleukin.
